# Equity-focused investment strategies to reduce maternal mortality in the Philippines: a subnational analysis using EQUIST

**DOI:** 10.7189/jogh.16.04360

**Published:** 2026-10-02

**Authors:** Krista Whitley, Polina Lebedeva, Andre Craig Christie, Annabelle Sim, Desere Thompson, Sally Hukpati, Lakshmi Balaji

**Affiliations:** 1 Department of Global and Environmental Health, School of Global Health, New York University, New York, New York, USA; 2 Department of Emergency Medicine, Kirk Kerkorian School of Medicine at UNLV, Las Vegas, Nevada, USA; 3 Trinity School of Medicine, Department of Medicine, Warner Robins, Georgia, USA

**Keywords:** maternal mortality, health equity, Philippines, EQUIST, maternal health, cost-effectiveness

## Abstract

**Background:**

Despite sustained national progress in maternal and child health, maternal mortality in the Philippines remains uneven across regions and socioeconomic groups. Women in poor, rural, and conflict-affected areas experience substantially higher maternal mortality than those in higher-income and urban populations. We aimed to assess subnational inequities in maternal mortality and estimate the potential impact and cost-effectiveness of prioritised maternal health interventions in Philippine regions with maternal mortality ratios exceeding the national benchmark.

**Methods:**

We conducted an equity analysis and developed an investment case using the UNICEF Equitable Impact Sensitive Tool (EQUIST). Regional maternal mortality ratios were based on 2019 subnational estimates, with the 2017 national maternal mortality ratio used as the benchmark for regional stratification. Intervention effectiveness estimates were drawn from published evidence syntheses and global clinical guidelines, and EQUIST’s costing module draws on LiST and the Marginal Budgeting for Bottlenecks approach. Three intervention packages were modelled: upgrading basic and comprehensive emergency obstetric and newborn care facility readiness; ensuring universal availability of uterotonics and magnesium sulphate; and expanding access to modern contraception among adolescents, women from poor households, and high-parity women. Projected maternal deaths averted, disability-adjusted life years averted, and cost per DALY were estimated through 2030.

**Results:**

Our modelling suggested that prioritised intervention packages could avert approximately 1,000–2,500 maternal deaths cumulatively through 2030. Facility readiness upgrades were projected to avert 1,000–1,500 maternal deaths, life-saving commodities 1,500–2,000, and modern contraception expansion 2,000–2,500. Sensitivity analysis varying key inputs by ±15% produced corresponding sensitivity ranges of 850–1,750, 1,275–2,300, and 1,700–2,875 maternal deaths averted, respectively. Modern contraception expansion had the lowest modelled cost per DALY averted at approximately US$150, compared with USD 300 for life-saving commodities and USD 450 for facility readiness upgrades.

**Conclusions:**

Maternal mortality in the Philippines remains concentrated among disadvantaged subnational populations. Equity-targeted investments, combined with strengthened emergency obstetric care, reliable life-saving commodities, and expanded access to modern contraception, can achieve substantial mortality reductions at highly cost-effective levels.

The Philippines has achieved measurable gains in maternal and child health over the last two decades. The 2022 National Demographic and Health Survey reported that, among women with a live birth in the preceding two years, 83.0% had four or more antenatal care visits, 89.6% of births were attended by a skilled provider, and 75.4% of women received a postnatal check within two days of delivery [1]. However, this national progress conceals persistent subnational inequities. The United Nations Maternal Mortality Estimation Inter-Agency Group estimated the maternal mortality ratio at 121 deaths per 100,000 live births in 2017, corresponding to approximately 2,700 maternal deaths [2].

Nevertheless, regional disparities remain pronounced. The Bangsamoro Autonomous Region in Muslim Mindanao (BARMM) reports substantially lower facility delivery and modern contraceptive use than national averages, while regions such as Eastern Visayas and Caraga have elevated reported maternal mortality, underscoring persistent geographic inequities in maternal health [1,3].

Although institutional delivery coverage is high nationally, important geographic inequities remain. The 2022 National Demographic and Health Survey reported that 88.4% of live births occurred in health facilities; the corresponding proportions were 91.8% among urban residents and 85.0% among rural residents [1]. BARMM remained a major outlier, with only 39.1% of live births occurring in health facilities [1]. Modern contraceptive use among currently married women was 41.8% nationally but only 20.5% in BARMM [1]. These disparities demonstrate substantial geographic differences in access to maternal and reproductive health services and provide important context for an equity-focused investment strategy.

These reproductive health inequities are also relevant to adolescents, a priority population in the contraception expansion package evaluated in this study. The 2022 National Demographic and Health Survey estimated an adolescent birth rate of 25 per 1,000 women aged 15–19 years [1]. This continuing burden provides additional rationale for examining equitable access to effective contraception among adolescents and other populations at increased risk of unintended pregnancy.

Although existing national surveys, mortality estimates, and subnational analyses document geographic disparities in maternal health outcomes and service coverage in the Philippines, they do not directly evaluate how limited resources could be prioritised across maternal health interventions to maximise health impact while addressing regional inequities. To address this gap, we applied EQUIST to compare the projected impact and cost-effectiveness of equity-focused maternal health intervention packages in Philippine regions with maternal mortality ratios exceeding the national benchmark [4].

## METHODS

We assessed subnational disparities in maternal mortality and service coverage and developed an equity-focused investment case using the United Nations Children’s Fund’s EQUIST, version 4.1 [4]. Within this analysis, we modelled the potential impact and cost-effectiveness of prioritised maternal health interventions in Philippine regions with maternal mortality ratios above the national average, estimating maternal deaths averted, disability-adjusted life years (DALYs) averted, and cost per DALY under alternative scale-up scenarios through an analytical time horizon of 2030.

The analysis included all 17 administrative regions of the Philippines. Regional maternal mortality inputs were based on 2019 mean maternal mortality ratio estimates reported by Ganancial *et al*. [3]. The 2017 national maternal mortality ratio of 121 deaths per 100,000 live births from the United Nations Maternal Mortality Estimation Inter-Agency Group was used as the national benchmark [2]. Regions were stratified according to maternal mortality burden relative to this benchmark, with high-burden regions defined as those exceeding the national maternal mortality ratio. The modelling framework explicitly applied an equity lens by prioritising scale-up in regions with higher mortality to maximise mortality reduction per million USD invested through 2030

To account for the deterministic nature of the EQUIST framework and assess the robustness of our projections, we conducted a manual parameter sensitivity analysis, whereby key model inputs – specifically, intervention effectiveness and target coverage thresholds – were varied by ±15. The resulting lower and upper bounds were reported as sensitivity ranges for projected maternal deaths averted and DALYs averted; these ranges represent deterministic scenario bounds rather than statistical confidence or credible intervals.

### Data sources and analytical assumptions

We derived the regional maternal mortality ratios used for regional stratification from the 2019 estimates reported by Ganancial *et al*. [3] and used the 2017 national maternal mortality ratio reported by the United Nations Maternal Mortality Estimation Inter-Agency Group as the national benchmark [2]. Global Burden of Disease 2019 data were used to contextualise the distribution of major causes of maternal mortality [5]. Intervention effectiveness parameters were drawn from Disease Control Priorities, 3rd edition [6] and World Health Organization clinical recommendations for the prevention and management of postpartum haemorrhage and hypertensive disorders of pregnancy [7,8]. Cost inputs were derived from published global unit cost estimates adjusted to 2020 USD, with standardised international costing assumptions applied where Philippine-specific cost data were unavailable.

We mapped DALY calculations within EQUIST directly from standard GBD assumptions, using a standard life expectancy baseline without age-weighting. For cost comparisons, EQUIST’s costing module draws on LiST and the Marginal Budgeting for Bottlenecks (MBB) approach [4]. A 3% annual discount rate was applied to both costs and health outcomes, consistent with the iDSI Reference Case for Economic Evaluation [9]. The analytical time horizon extended to 2030 to align with national planning cycles and Sustainable Development Goal targets.

### Modelled intervention packages

Maternal mortality in the Philippines is primarily driven by preventable obstetric complications among women of reproductive age (15–49 years), with postpartum haemorrhage, hypertensive disorders of pregnancy including preeclampsia and eclampsia, sepsis, and complications from unsafe abortion accounting for the majority of deaths [5]. Estimates from the Global Burden of Disease Study 2019 indicate that postpartum haemorrhage remains the leading cause, followed by hypertensive disorders and infection-related complications [5]. The intervention packages selected for this analysis are directly aligned with these predominant causes of mortality.

We identified three prioritised intervention packages for inclusion in our model for developing the investment case. The first of these was a facility readiness upgrade package, which included the expansion and strengthening of basic emergency obstetric and newborn care (BEmONC) and comprehensive emergency obstetric and newborn care (CEmONC) capacity, including staffing, equipment, operating theatre capability, and blood transfusion availability. This package strengthened BEmONC and CEmONC capacity to manage obstetric emergencies including severe bleeding, infection, and obstructed labour. The second intervention package was a life-saving commodity package, which comprised universal availability and administration of uterotonics for prevention and treatment of postpartum haemorrhage and magnesium sulphate for management of severe pre-eclampsia and eclampsia. This package targets postpartum haemorrhage and eclampsia through the scale-up of uterotonics and magnesium sulphate. The final, third package was a modern contraception expansion package, which included scale-up of modern contraceptive access among adolescents, women in poor households, and high-parity women to reduce unintended and high-risk pregnancies, thereby lowering overall exposure to maternal mortality risk in high-burden regions.

Our model assumed proportional mortality reduction corresponding to intervention effectiveness estimates from published meta-analyses and global clinical guidelines [6–8]. Coverage expansion scenarios modelled incremental scale-up from baseline to target coverage thresholds consistent with the Philippine Health Sector Strategy 2023–2028 [10]. These scenarios were considered alongside the UNFPA Country Programme target of reducing maternal mortality to 74 per 100,000 live births by 2028 [11]. Equity impact was assessed by comparing projected mortality reductions across regions and quantifying gains in high-burden regions relative to the national benchmark.

## RESULTS

Regional stratification identified Caraga (145.79 maternal deaths per 100,000 live births), Eastern Visayas (138.56), and Cagayan Valley (128.56) as exceeding the 2017 national benchmark of 121, whereas BARMM (52.77) was classified below the benchmark (**Table 1**, **Table 2**; Figure S1 in the **Online Supplementary Document**).

**Table 1:** Regional maternal mortality ratios per 100,000 live births, Philippines, 2019, and classification relative to national average

Region	Maternal mortality ratio per 100,000 live births	Classification relative to national average (120–125)
BARMM	52.77	Below national average (lower burden)
Eastern Visayas	138.56	Above national average (high burden)
Caraga (Region XIII)	145.79	Above national average (high burden)
Cagayan Valley	128.56	Above national average

BARMM – Bangsamoro Autonomous Region in Muslim Mindanao

**Table 2:** Major causes of maternal mortality in the Philippines and corresponding intervention packages

Major cause of maternal mortality	Current intervention package
Hypertensive disorders (pre-eclampsia/eclampsia)	Magnesium sulfate + facility readiness
Postpartum haemorrhage	Uterotonics + blood supply + CEmONC
Pregnancy with abortive outcome	Modern contraception expansion
Sepsis/infection	BEmONC/CEmONC + antibiotics
Obstructed labor	Facility upgrade + referral systems

BEmONC – basic emergency obstetric and newborn care, CEmONC – comprehensive emergency obstetric and newborn care

Our baseline modelling projected that the intervention packages could avert approximately 1,000–2,500 maternal deaths cumulatively through 2030. Specifically, facility readiness upgrades were projected to avert 1,000–1,500, life-saving commodities 1,500–2,000, and modern contraception expansion 2,000–2,500 maternal deaths (**Figure 1**; **Table 3**). Sensitivity analysis varying key inputs by ±15% produced ranges of 850–1,750 deaths averted for facility readiness upgrades, 1,275–2,300 for life-saving commodities, and 1,700–2,875 for modern contraception expansion.

**Figure 1:**
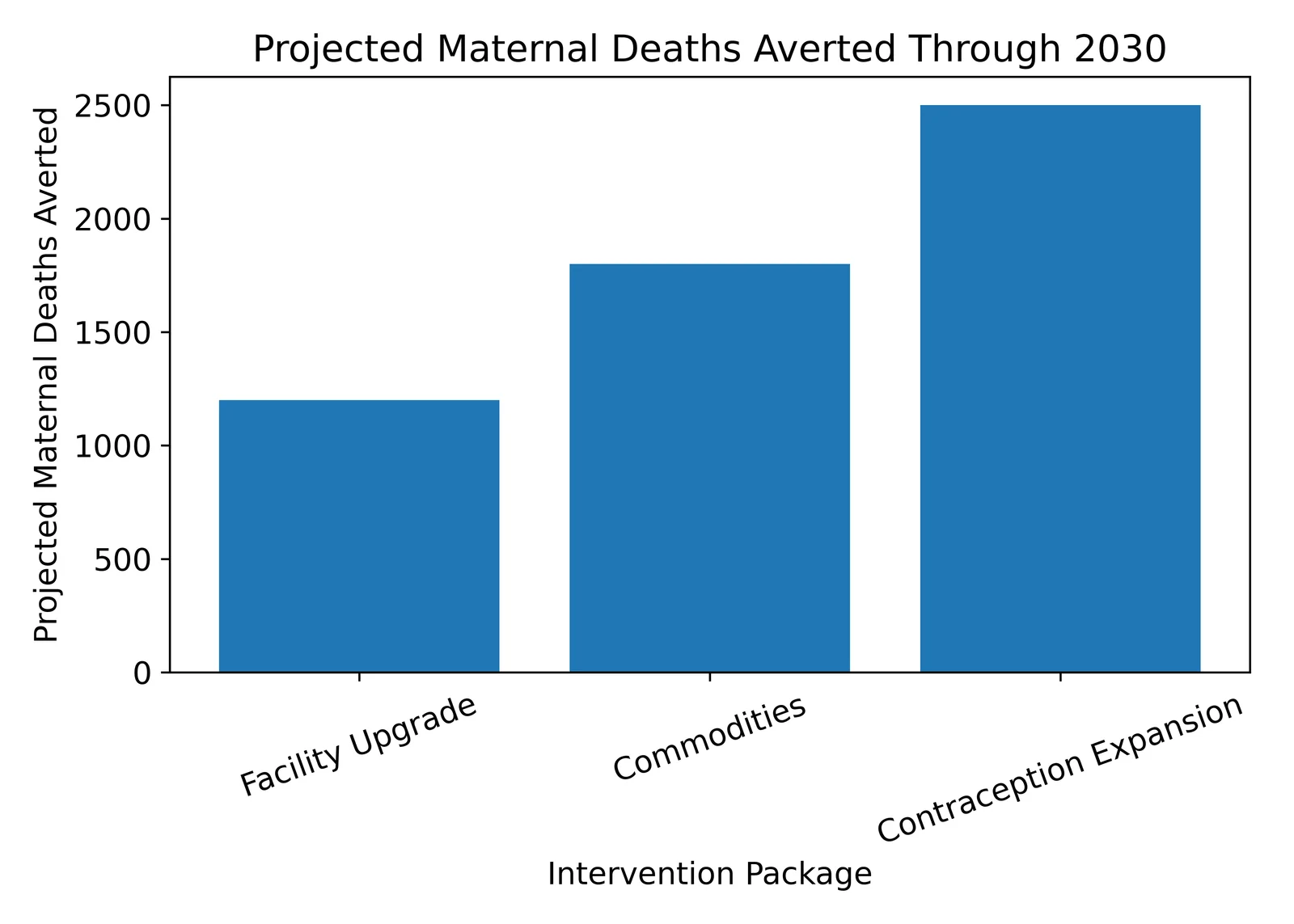
Intervention impact comparison. Projected maternal deaths averted by intervention package through 2030. Facility readiness upgrades are estimated to avert approximately 1000–1500, life-saving commodities approximately 1500–2,000, and modern contraception expansion approximately 2000–2,500 maternal deaths cumulatively through 2030.

**Table 3:** Summary of modelled outcomes by intervention package, including projected maternal deaths averted with ±15% sensitivity ranges, relative resource requirements, relative cost per DALY averted, and relative cost-effectiveness

Intervention package	Projected maternal deaths averted, cumulative, through 2030 (±15% sensitivity range)	Relative resource requirements	Relative cost per DALY averted	Relative cost-effectiveness
Facility readiness upgrade (BEmONC/CEmONC)	1,000–1,500 (850–1,750)	High (capital-intensive)	Higher relative cost per DALY	Moderate
Life-saving commodities (uterotonics + magnesium sulfate)	1,500–2,000 (1,275–2,300)	Low to moderate	Low	High
Modern contraception expansion	2,000–2,500 (1,700–2,875))	Moderate	Moderate	Very high

BEmONC – basic emergency obstetric and newborn care, CEmONC – comprehensive emergency obstetric and newborn care, DALY – disability-adjusted life years, UI – uncertainty interval

Cost-effectiveness also differed across the three packages. Modern contraception expansion had the lowest modelled cost per DALY averted at approximately USD 150, compared with USD 300 for life-saving commodities and USD 450 for facility readiness upgrades (**Figure 2**). The cost per DALY averted for modern contraception expansion was therefore approximately 50% lower than for life-saving commodities and 67% lower than for facility readiness upgrades; life-saving commodities were approximately 33% lower than facility readiness upgrades.

**Figure 2:**
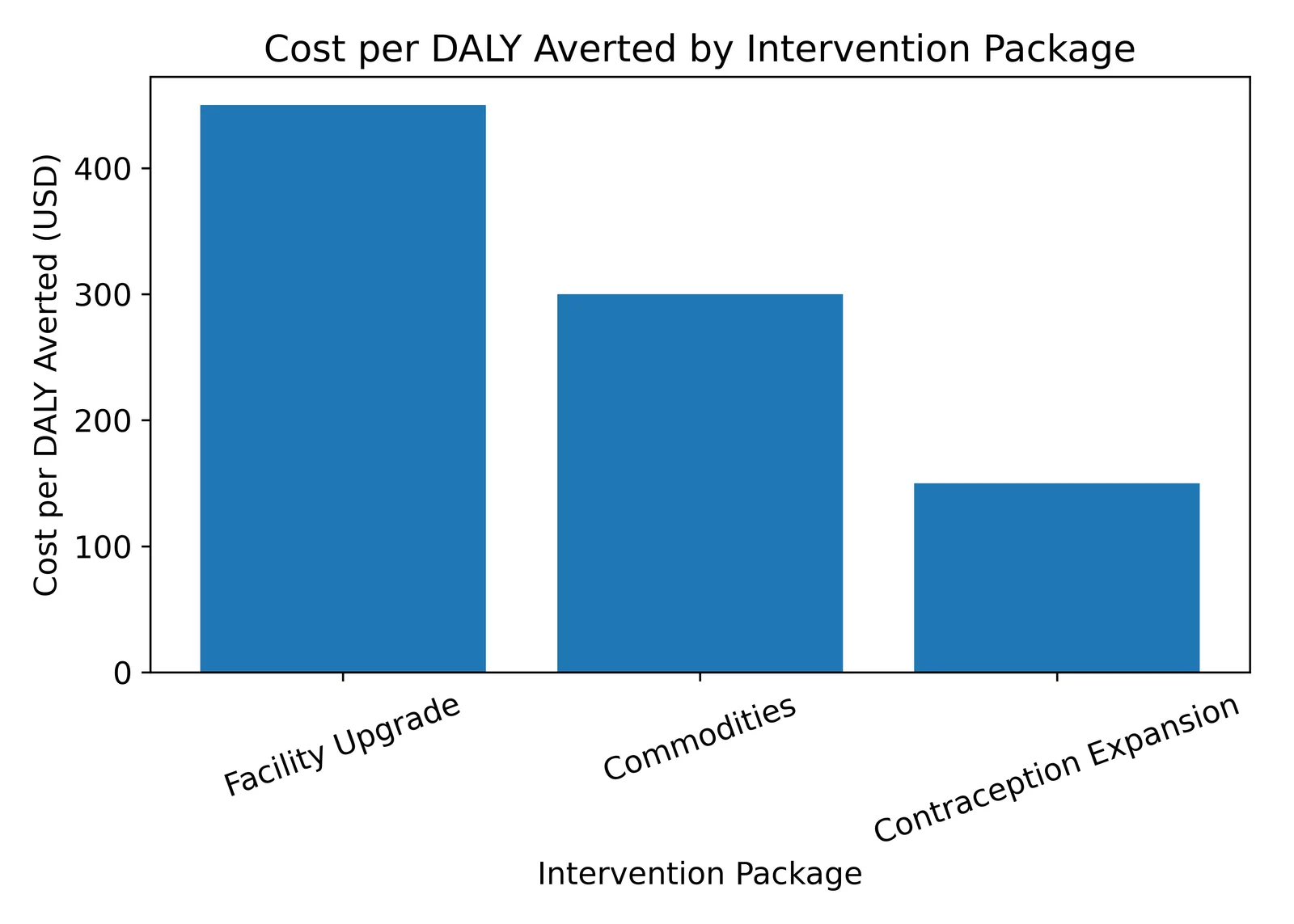
Cost-effectiveness comparison. Cost per DALY averted in USD by intervention package. Facility readiness upgrades have an estimated cost of approximately USD 450, life-saving commodities approximately USD 300, and modern contraception expansion approximately USD 150 per DALY averted, indicating the highest cost-effectiveness for contraception expansion.

Equity-targeted allocation concentrated the projected mortality benefit in high-burden regions (**Figure 3**). Facility readiness upgrades were projected to avert approximately 900 maternal deaths in high-burden regions compared with 300 in lower-burden regions; the corresponding estimates were approximately 1,300 vs 500 for life-saving commodities and 2,000 vs 500 for modern contraception expansion. Thus, high-burden regions accounted for approximately 75%, 72%, and 80% of the projected deaths averted under the facility readiness, life-saving commodity, and modern contraception packages, respectively. Across all three packages, most projected mortality reductions therefore occurred in high-burden regions under equity-targeted allocation.

**Figure 3:**
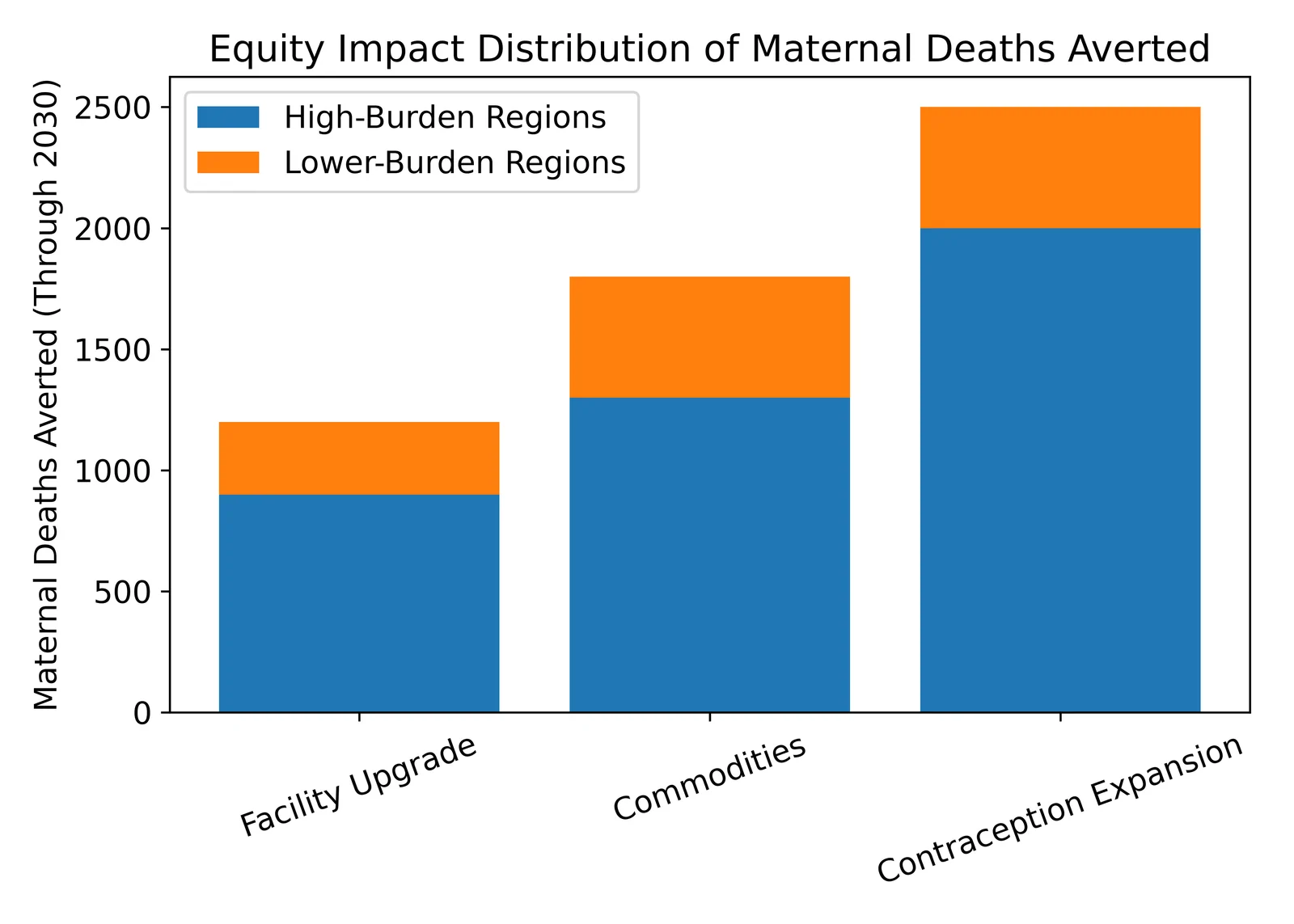
Equity impact distribution. Distribution of maternal deaths averted by intervention package across high-burden and lower-burden regions through 2030. Facility readiness: approximately 900 *vs*. 300 deaths averted. Commodities: approximately 1300 *vs*. 500 deaths averted. Contraception expansion: approximately 2000 *vs*. 500 deaths averted. This indicates that most impact occurs in high-burden regions.

## DISCUSSION

Through this analysis, we developed an equity-focused investment case and demonstrated that maternal mortality in the Philippines is less a question of the availability of effective interventions than of their timely and equitable delivery with sufficient quality. National maternal health indicators show relatively high aggregate contact coverage [1]. However, these gains have not eliminated preventable maternal mortality, suggesting that gaps in service quality, timeliness, and emergency response capacity remain important constraints. This pattern is consistent with recent national and partner planning frameworks that explicitly emphasise “leaving no one behind” as a central implementation challenge [1,11,12]. Evidence from facility assessments in the Philippines also indicates that emergency obstetric care capacity can remain incomplete despite broader gains in maternal service coverage. A facility assessment in Luzon found that only 35% of 245 BEmONC-provider rural health units were adequately functional, with personnel capacity contributing to lower overall functionality [13]. Earlier assessments following Typhoon Haiyan likewise documented shortages of life-saving commodities, including magnesium sulphate [14]. These findings support the need to distinguish 

Without targeted investment in high-burden regions, national averages risk masking severe local vulnerability. Although national institutional delivery coverage appears high, these averages obscure critical survival gaps in high-burden regions such as BARMM, where facility delivery remains substantially lower than the national average [1]. This means that many women still face childbirth complications outside settings capable of managing postpartum haemorrhage, eclampsia, obstructed labour, and sepsis, which are all conditions that require immediate emergency obstetric care. In these contexts, maternal mortality is driven less by whether women seek care and more by whether life-saving treatment is available quickly enough to prevent death. Without strengthened BEmONC and CEmONC readiness, improvements in antenatal care coverage alone will not translate into survival gains.

The persistently low contraceptive use in high-burden regions also reflects continued unmet needs for family planning, increasing the exposure to unintended pregnancies, unsafe abortion, and preventable maternal risk [1,15]. Failure to prioritise contraception expansion, therefore, sustains both immediate obstetric risk and long-term inequities in maternal survival. Modern contraception expansion demonstrates profound cost-effectiveness because it structurally drives down absolute maternal risk exposure. While safely averting complications tied to unsafe abortions, the intervention minimises total obstetric emergencies before they ever manifest at a facility level by preventing high-risk, high-parity, and adolescent pregnancies.

These findings support an equity-focused investment approach that prioritises the most vulnerable populations and directs resources towards areas where mortality burden and service gaps are greatest [4,16]. In this analysis, that principle translates into strengthening service readiness and access to modern contraception where baseline coverage and access are lowest, rather than relying on national averages that may mask regional vulnerability. A major paradox in the subnational data is the classification of BARMM as a lower-burden region despite low facility delivery and contraceptive access rates. This finding must be interpreted with extreme caution. The discrepancy between BARMM’s comparatively low reported MMR and its substantially lower service coverage warrants cautious interpretation and should not be assumed to represent a protective health effect.

Several components needed to reduce maternal mortality are already articulated in the Philippines’ planning architecture. The Philippine Health Sector Strategy 2023–2028 prioritises reducing preventable maternal deaths and strengthening primary health care systems [10]. These priorities are directly relevant to the service-readiness, referral, and equity gaps identified in our analysis.

The concentration of projected mortality reductions in high-burden regions (**Figure 3**) is consistent with the equity-focused allocation approach and provides the study-specific basis for interpreting these findings in relation to the ‘leave no one behind’ principle.

Furthermore, UNFPA’s Country Programme Document 2024–2028 [11] explicitly prioritises ending preventable maternal deaths and reducing unmet need for family planning, with particular attention to adolescents and populations in geographically isolated and disadvantaged areas. It also emphasises health system strengthening, quality improvement, and accountability mechanisms, as well as integrating demographic evidence into decision-making. These are aligned with the needs identified in our analysis, where high-burden regions require stronger facility readiness, reliable commodities, and effective referral systems to translate coverage into survival.

The UNFPA Country Programme Evaluation for 2019–2023 [12] defines where progress stalls and what must change in the next cycle. It highlights priority focus areas including quality of care and services, gender and social norms, and adolescents and youth, with population data mainstreamed across the programme. These priorities map directly onto the barriers reflected in maternal mortality: preventable deaths persist when the quality of intrapartum and emergency care is inadequate, when adolescent pregnancy and unmet need remain high, and when norms and access barriers prevent timely care-seeking. The Philippine Population and Development Plan of Action 2023–2028 emphasises modern contraception expansion and adolescent reproductive health [17].

Finally, decentralisation and financing reforms are now part of the operating environment. The Mandanas-Garcia ruling expanded fiscal transfers to local government units (LGUs) beginning in 2022 and shifted greater implementation responsibility to subnational actors [18]. This can support equity when investments are targeted to high-burden LGUs, but it can also widen gaps when technical capacity, procurement performance, and service readiness vary across LGUs. The policy environment therefore makes the ‘how’ of implementation – financing flows, accountability, and execution capacity – just as important as the ‘what’ of the intervention package

### Policy implications and operational recommendations

While the quantitative EQUIST modelling highlights the specific mortality reductions achievable through targeted clinical packages, translating these scenario projections into real-world survival gains requires addressing broader, structural health system functions. We propose some recommendations with broader policy interpretations aimed at addressing the cross-cutting systemic barriers identified in our analysis, though they extend beyond the direct mathematical outputs generated by the EQUIST.

Referral and transport systems must be operationalised as structural priorities, rather than passive add-ons. Many subnational maternal deaths are driven by inter-facility delays. Regions should define measurable benchmarks for referral response times and ambulance availability, particularly within geographically isolated and disadvantaged areas.

Upgraded facility readiness is functionally dependent on stable blood banking networks and emergency surgical staffing stability. Forward strategies must prioritise subnational procurement pipelines for cross-matching capacity and blood supply chains in district hospitals serving high-burden catchments.

To move from ‘contact coverage’ to ‘effective coverage’, quality improvement must be systematised *via* continuous facility readiness scorecards and robust maternal and perinatal death surveillance and response loops that tie audit findings directly to local budgeting actions.

Recognising that adolescent pregnancy and reproductive risks are deeply intertwined with psychosocial vulnerabilities, implementation strategies should explicitly integrate adolescent-friendly reproductive health services with mental health screenings, gender-based violence response pathways, and targeted HIV prevention.

In light of the Mandanas-Garcia ruling’s fiscal devolution, an urgent operational opportunity exists to build local government unit procurement, supply chain management, and fund absorption capacity. National technical support mechanisms should prioritise high-burden local government units to ensure increased fiscal space translates directly into equitable maternal health outcomes.

Given the Philippines’ extreme hazard exposure, maternal health delivery networks are frequently fractured by typhoons, flooding, and shifting displacement patterns. Reproductive health infrastructure planning must build explicit service continuity plans for transport networks, emergency staffing, and cold-chain integrity during climate shocks.

### Limitations

In general, EQUIST modelling relies on assumptions of uniform intervention effectiveness and does not fully capture local health system capacity constraints. Cost projections are static and do not account for macroeconomic changes. Behavioural determinants and sociopolitical barriers are not directly modelled. These limitations suggest that our results should be interpreted as scenario projections, rather than predictive outcomes.

## CONCLUSIONS

Maternal mortality in the Philippines remains unacceptably high in disadvantaged subnational regions despite the availability of effective and affordable interventions. Equity-targeted investments that prioritise emergency obstetric care readiness, life-saving commodities, and expanded access to modern contraception can substantially reduce maternal deaths at highly cost-effective levels. Focused resource allocation to high-burden regions is both epidemiologically justified and economically efficient, and is essential for achieving national and global maternal health targets.

## Additional material


Online Supplementary Document

## Data Availability

**Data availability:** No new primary data were collected. The data and model inputs supporting the analysis are available from the cited sources and the publicly available EQUIST platform and documentation.
