# Supplementary material for: Equity-focused investment strategies to reduce maternal mortality in the Philippines: a subnational analysis using EQUIST
Source: J Glob Health. 2026 Oct 2;16:04360. doi: 10.7189/jogh.16.04360 (PMC13631932; doi:10.7189/jogh.16.04360)
Supplement: Supplementary file 1 [file jogh-16-04360-s001.pdf]

Supplement to: Whitley K, Lebedeva P, Christie AC, Sim A, Thompson D, Hukpati S, Balaji L. Equity-focused investment strategies to reduce maternal mortality in the Philippines: a subnational analysis using EQUIST. J Glob Health. 2026;16:04360.

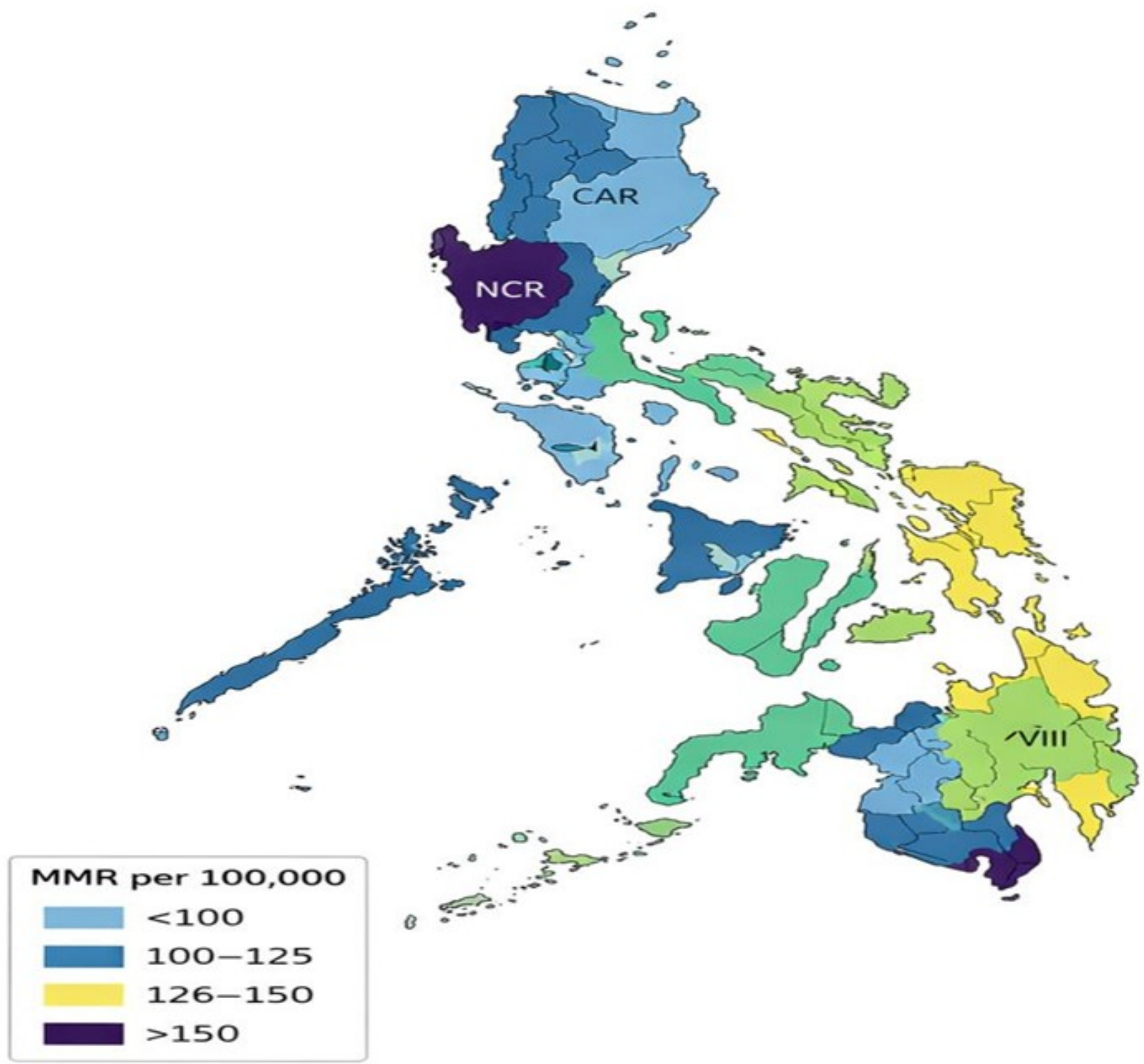

Figure S1. Geographic distribution of maternal mortality ratios by region, Philippines, 2019. Source: Authors' analysis of 2019 regional maternal mortality estimates reported by Ganancial et al. [15].
